# The Role of Interleukins in Pediatric Traumatic Brain Injury: A Narrative Synthesis

**DOI:** 10.3390/jcm15010186

**Published:** 2025-12-26

**Authors:** Christodoulos Komiotis, Ioannis Mavridis, Efstratios-Stylianos Pyrgelis, Eleni Agapiou, Maria Meliou, Theodossios Birbilis

**Affiliations:** 1 School of Medicine, Democritus University of Thrace, 681 00 Alexandroupolis, Greece; 2 Department of Neurosurgery, University General Hospital of Alexandroupolis, 681 00 Alexandroupolis, Greece

**Keywords:** pediatric traumatic brain injury, interleukins, cytokines, neuroinflammation

## Abstract

Traumatic brain injury (TBI) is a common and important cause of morbidity and mortality among pediatric patients, affecting 47–280 per 100,000 children every year. Head trauma can affect the brain not only by the injury itself but also via a neuroinflammatory process, which leads to blood–brain barrier (BBB) disruption, leukocyte infiltration, and edema formation. This process is regulated by several immune mediators, including interleukins (ILs), which are molecules that are currently investigated in both adult and pediatric TBI. In pediatric patients, IL-1β, IL-6, and IL-8 have mainly been investigated, while IL-10 and IL-17 also play a role in the neuroinflammatory cascade. Therefore, the purpose of this review was to examine the role of the aforementioned cytokines in the pathophysiology of pediatric TBI, as well as their role in determining clinical outcome and prognosis. IL-1β is a key pro-inflammatory cytokine in glutamate excitotoxicity post-TBI and in upregulating the expression of additional pro-inflammatory cytokines. Its high levels in cerebrospinal fluid (CSF) are correlated with injury severity and poor outcomes. IL-6 is an anti-inflammatory cytokine, and its concentration rises rapidly after the injury. Current data show that it can be useful in predicting severe TBI (sTBI) in addition to clinical parameters. IL-8 is a cytokine with several pro- and anti-inflammatory properties. On the one hand, it is a potent chemotactic agent, attracting inflammatory cells to the injured area, and it plays a role in BBB disruption. On the other hand, it promotes the survival of cholinergic and hippocampal neurons via the secretion of nerve growth factor (NGF). These cytokines are important in predicting the outcome of pediatric patients with TBI, as well as in predicting several post-TBI conditions such as fatigue and epilepsy, thus improving diagnostic ability and timely treatment. Further research, unraveling the complex mechanisms via which post-TBI neuroinflammation occurs, will lead to targeted therapies and better outcomes overall.

## 1. Introduction

Traumatic brain injury (TBI) remains the leading cause of morbidity and mortality among pediatric patients worldwide, affecting 45 per 100,000 children every year [1], and can be classified as mild, moderate, or severe, based mainly on clinical criteria, including loss of consciousness, alteration of mental state, post-traumatic amnesia, and Glasgow Coma Scale score [2]. Today, additional criteria such as imaging and pathoanatomical measures also affect classification [3]. TBI arises due to mechanical force that leads to a temporary or permanent impairment of brain function (with an altered state of consciousness, physical, psychological, and cognitive dysfunction) and causes brain damage in a primary and a secondary way. In the primary way, damage is caused by the injury itself, while in the secondary way, the blood–brain barrier (BBB) is disrupted, and this causes leukocyte infiltration, cerebral edema, and production of many immune mediators, including interleukins (ILs) [4].

TBI provokes an acute inflammatory response that contributes significantly to the pathophysiology of secondary damage after TBI. This inflammatory response is caused as a result of a sequence of molecular processes, where pro-inflammatory cytokines, anti-inflammatory cytokines, and chemokines play a crucial role. They are directed to the site of the injury and interact with neurons, microglia, oligodendrocytes, and astrocytes [5,6].

The initial trauma after TBI is followed by a complex cascade of cellular events causing tissue damage and dysregulation of cerebral blood flow (CBF) and cerebral metabolism. Moreover, the disruption of blood vessels leads to vasogenic brain edema and the intracellular accumulation of water to cytotoxic brain edema. During these processes inflammatory cytokines are released, leading to cell death [7,8].

In children, TBI exhibits a bimodal age distribution, with children younger than 2 or older than 15 years being mostly affected. Mild TBI accounts for the majority of injuries [9]. Additionally, assessment of pediatric patients is challenging since repetitive radiologic evaluation and invasive monitoring of intracranial pressure (ICP) are more difficult than in adults. Using biomarkers based on simple, rapid blood or cerebrospinal fluid (CSF) tests instead could provide information on the extent of brain damage and potentially predict clinical outcomes [10].

Cytokines, for instance, are known to play an important role in mediating the inflammatory response provoked within the central nervous system (CNS) following TBI [11]. Some of the most important biochemical mediators are pro- and anti-inflammatory ILs, specifically IL-1, IL-6, IL-8, and IL-10. These are measured using an Enzyme-linked Immunosorbent Assay (ELISA) in either blood or CSF samples [5,12].

Therefore, the goal of this review is to analyze how these mediators are involved in the pathophysiology and clinical severity of TBI, as well as how their levels can affect the diagnosis, prognosis, and therapy of children suffering from TBI.

## 2. Methods

Methodologically, we performed a search in the PubMed/Medline database using the terms “traumatic brain injury”, “children”, and “interleukin”, which retrieved 77 results (until May 2024). The references of the retrieved articles were also examined for additional potentially eligible studies. Title and abstract screening was performed by four independent investigators, and minor disagreements were assessed through discussion. Full text review was then performed.

**Figure 1:**
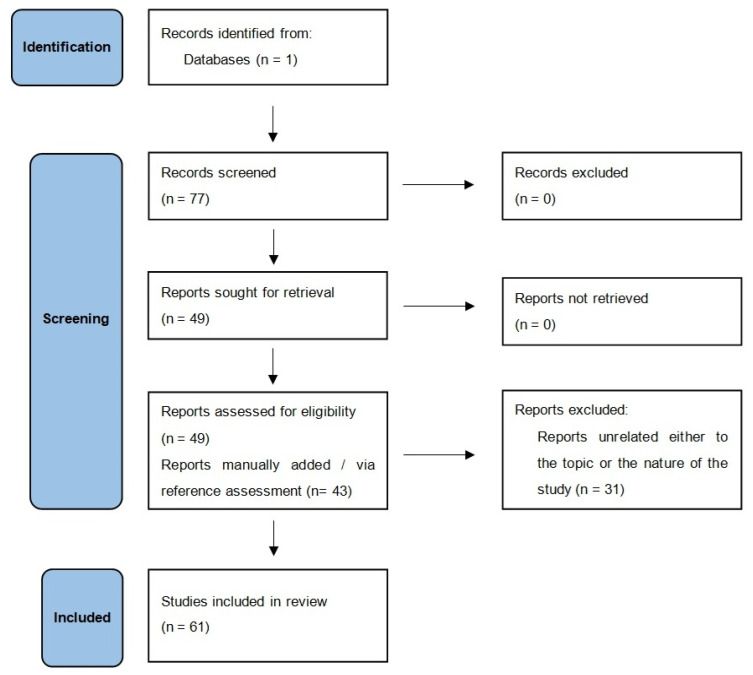
Study selection flowchart.

Original research publications examining the role of IL-1β, IL-6, IL-8, IL-10, and IL-17 in pediatric (age < 18 years) patients with mild, moderate, or severe TBI met our inclusion criteria, whereas animal studies were excluded. Non-original studies were only considered if they examined the pathophysiological mechanisms via which ILs were involved in pediatric TBI. After careful investigation and exclusion of non-relevant articles, we finally used 44 articles for our review. Additionally, while the presentation of findings is narrative, the methodology follows scoping review standards. A flow diagram illustrating our study selection process (modified from the Preferred Reporting Items for Systematic reviews and Meta-Analyses (PRISMA) statement [13]) can be found in Figure 1.

## 3. Pathophysiology of Pediatric Traumatic Brain Injury

There are both acute and chronic pathophysiological changes that can affect the developing brain of children with TBI. Firstly, the injury itself can damage the brain due to the mechanical contact that occurred at the moment of impact. Secondly, the brain can be damaged by the pathophysiological changes that occur in response to tissue injury and have delayed clinical presentation [14,15].

More precisely, CBF changes occur after the injury (mostly hypoperfusion, but hyperperfusion is also seen), as well as failure in cerebrovascular autoregulation, which is irrespective of the severity of injury [14,15]. Another important post-TBI phenomenon is vasospasm, which is also a prognostic factor for outcomes. Additionally, there are changes in cerebral metabolism and glucose and oxygen consumption, and these can be predictive of outcomes as well. Most often patients have lower metabolic rates (although hypermetabolism of glucose has also been observed), which is caused by mitochondrial dysfunction. Moreover, a massive release of excitatory neurotransmitters has been reported, as well as reactive oxygen species (ROS) production, which, in turn, leads to oxidative stress. All these changes, together with other mediators secreted by glial cells -such as ILs- lead to edema and inflammation [14,15].

As stated earlier, primary and secondary insults lead to the release of soluble mediators such as cytokines, chemokines, prostaglandins, free radicals, and complement, all of which regulate the pathophysiological changes that occur over time. This is where ILs are important; these mediators are important in modifying the BBB, increasing vascular permeability, thus mediating edema formation and leukocyte extravasation, and they also act as potent chemotactic agents attracting other leukocytes [16].

## 4. ILs and Their Role in Pediatric TBI

### 4.1. Overview of ILs

Neuroinflammation is a core element of secondary brain damage following TBI. This process is regulated by several mediators, including ILs, which seem to play an important role in the pathophysiology of TBI.

In summary, IL-1β, IL-4, and IL-12 are the main pro-inflammatory ILs that are indicative of neuroinflammation and have been associated with neurotoxicity and neuronal damage, whereas IL-6, IL-8, and IL-10 seem to have neuroprotective activity. These cytokines contribute to the biosynthesis of neurotrophins such as nerve growth factor (NGF), brain-derived neurotrophic factor (BDNF), and glial-derived neurotrophic factor (GDNF), which, in turn, play a crucial role in neuronal recovery [17].

**Figure 2:**
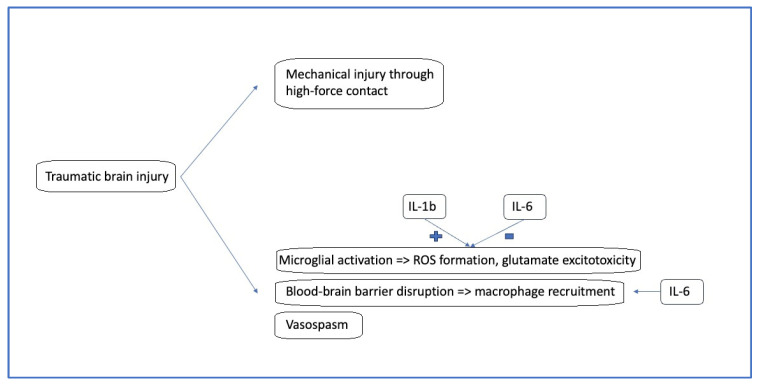
Simplified pathophysiology of pediatric TBI. IL, interleukin; ROS, reactive oxygen species.

Additionally, ILs are a group of cytokines that play an essential role in activating and regulating the immune system. They were initially believed to be expressed by leukocytes; however, it is now known that they can be produced by many types of cells. Finally, ILs, whether pro- or anti-inflammatory, are important in promoting growth, differentiation, and activation of immune cells during inflammation, as well as in terminating the immune response [18,19,20,21]. The pathophysiology of pediatric TBI is summarized in Figure 2.

### 4.2. Specific ILs and Their Roles in Pediatric TBI

#### 4.2.1. IL-1β

IL-1β is one of the most important pro-inflammatory cytokines, and it is mainly secreted by microglia, although it can also be secreted by damaged endothelial cells and astrocytes. IL-1β is expressed both in moderate and severe TBI, mainly during the acute phase, and exacerbates neuronal damage by promoting inflammation and high temperature. Its levels have also been associated with the severity of the injury and poorer outcomes [11,22].

When it comes to its function in the nervous system, it is involved in glutamate excitotoxicity and loss of oligodendrocytes via apoptosis [23], and also in the production of prostaglandins, which are key mediators of inflammation [22]. Apart from that, IL-1β acts synergistically with the neurotoxic tumor necrosis factor alpha (TNFα), leading to the release of nitric oxide synthase, free oxygen radicals, and excitatory aminoacids [11,22,24].

In TBI studies, CSF IL-1β levels rise slowly in the first two hours post-injury and continue to rise for the next 48 h, although this rise is not as dramatic as the rise in IL-6 [12]. Then, IL-1β levels continue to rise, in contrast to other biomarkers, which tend to decline, thus implicating the start of the neuroinflammatory cascade. The inflammatory response induced by head trauma contributes to secondary brain injury through cytokine production, complement activation, and neurotrophic infiltration. An early increase in IL-1β levels is associated with severe head injury and poor neurological outcomes [17,25].

#### 4.2.2. IL-6

IL-6 plays a major role in both inflammatory and healing processes of TBI. As described in 2015, IL-6 is produced by astrocytes when they undergo mechanical or metabolic injury, although it can also be produced by neurons. It is an important signaling molecule in the central nervous system (CNS), and it plays an important role in synaptic transmission and synaptic plasticity, which are important for memory and learning. It could be an important regulator of CNS pathways that are critical to cognitive function, although further studies in humans are needed to confirm this [26].

In TBI the membrane of brain cells is disturbed, leading to the release of IL-6, along with other neuronal markers, such as neuron-specific enolase (NSE), into circulation, where it regulates activities of mononuclear cells and participates in glial regeneration and the production of neurotrophins, such as NGF [17,27]. Therefore, along with NGF, it plays a neuroprotective role in the damaged brain by promoting cholinergic neuron survival and supporting additional recovery mechanisms, and its expression is mainly associated with favorable outcomes [11,17,28].

In several studies with pediatric TBI patients, however, the results regarding IL-6 are controversial. In one study, IL-6 levels on admission were slightly lower in the group with unfavorable outcomes. One week later this biomarker had various levels and was either increased or decreased. In the same study, in children with mild TBI, all biomarkers for brain damage were lower, and one week after trauma, a decrease was observed. According to the authors, the initial serum levels of IL-6 were not correlated with the severity of brain damage [9]. However, in another study, IL-6 levels were significantly elevated in patients with TBI and could also predict increased ICP. Therefore, the authors proposed IL-6 as a useful biomarker in TBI to identify those at risk of increased ICP [29]. These results, however, were observed in patients with isolated TBI, without additional injuries. In a different study, an increase in both IL-1β and IL-6 was correlated with TBI severity and poor clinical outcome [25]. Finally, in a study with both adult and pediatric TBI patients, IL-6 as well as IL-10 were increased in both groups and were also associated with increased injury severity and poorer outcomes [30].

Finally, in pediatric patients with severe TBI, IL-6 levels, similarly to IL-1β, rapidly rise in the CSF within two hours post-injury [17].

#### 4.2.3. IL-8

IL-8 is a potent chemoattractant for neutrophils and is a member of the C-X-C chemokine ligand family (CXCL8), a group of signaling molecules that act as biological markers of the inflammatory response to injury, and is secreted by glial cells, macrophages, and endothelial cells [31]. Apart from its function in the cells of the immune system, IL-8 is shown to be associated with BBB dysfunction, as well as to increase NGF after injury and to promote the survival of cholinergic and hippocampal neurons, as Kossman et al. [32] describe.

This cytokine is also an important inflammatory chemokine, with chemotactic and neutrophil-activating abilities. When it is traced in the CSF of patients with severe TBI, it has been correlated with higher mortality [1,33]. IL-8 has been found to be markedly increased in the CSF of pediatric patients with TBI within 12 h following injury, and its levels remain high for up to 108 h [33]. Finally, contrary to its pro-inflammatory properties, IL-8 also regulates several regenerative processes, leading to post-injury healing [33].

#### 4.2.4. IL-10

IL-10 is a key anti-inflammatory cytokine that is produced within the CNS, and its role is to limit the severity of a spectrum of vascular, neurodegenerative, and infectious conditions. Specifically, it promotes neuronal and glial cell survival by blocking the effects of other pro-inflammatory cytokines, including TNFα, IL-1, IL-6, IL-8, and interferon gamma (INFγ), and promoting the expression of cell survival signals. It decreases the release of ROS and nitric oxide [27] and interacts with different pathways, including Janus kinase-signal transducer and activator of transcription (Jak1/Stat3), phosphoinositide 3-kinase (PI3K), mitogen-activated protein kinase (MAPK), suppressor of cytokine signaling (SOCS), and nuclear factor kappa B (NFκB), having a regulatory effect on them. It further downregulates the expression of other ILs’ receptors and blocks their activation [34]. In TBI experimental models, IL-10 has been shown to alleviate the inflammatory cascade that is induced after injury and, thus, promotes recovery [35].

According to a study by Tsitsipanis, serum IL-10 levels were higher in the group with severe TBI and in those with poorer long-term outcomes. This study, however, included adult and pediatric patients [30].

#### 4.2.5. IL-17

Regarding IL-17, its role in the CNS has mainly been investigated in animal models. In multiple sclerosis models, for example, higher levels of IL-17 were associated with disease severity and progression. Additionally, IL-17 has been linked to impaired BBB integrity and microglial activation [36]. Finally, it has also been found to regulate different pathways, such as NFκB, and to promote glutamate excitotoxicity [37]. Table 1 summarizes the main findings regarding the key ILs in pediatric TBI.

## 5. Findings from Recent Clinical Studies

Ryan et al. [39] performed a cytokine analysis, measuring the plasma levels of IL-2, IL-4, IL-6, IL-8, IL-10, and IL-17A, among other cytokines, in children with severe [Glasgow Coma Scale (GCS) ≤ 8/15] and mild (GCS = 14/15) TBI (sTBI, mTBI). Their study involved 208 children: 104 with mTBI, 6 with sTBI, and 98 controls. At the time of injury, IL-6 was raised in every patient, with significantly higher levels among patients with sTBI, and a cutoff value of 20 pg/mL IL-6 was 100% sensitive and 99% specific for sTBI, and 91% sensitive but not specific for mTBI. Interestingly, in children with mTBI, IL-8, IL-10, and IL-17A were decreased, although they remained unchanged in children with sTBI. Differences in IL-2 and IL-4 were not statistically significant (*p* > 0.05), and differences among genders were also not significant (*p* > 0.05). In terms of diagnosing mTBI and sTBI, the authors were able to identify sTBI patients with 100% sensitivity and specificity (area under the curve (AUC) = 1) using IL-6, while for mTBI, they observed that the ratio of IL-6/IL-8 had the best results (AUC = 0.829).

Crichton et al. [38] tried to describe the best combination of biomarkers that could predict fatigue, one of the most common post-TBI symptoms, 12 months post-injury, in children. Their study involved 87 children with TBI: 50 with mTBI (GCS = 13–15), 8 with moderate TBI (GCS = 9–12), and 29 with sTBI (GCS < 9). They measured six serum biomarkers within 24 h from injury: IL-6, IL-8, S100 calcium binding protein B (S100B), NSE, and soluble neural cell adhesion molecule (sNCAM). Based on their results, they claimed that IL-8 has the best prognostic value following pediatric TBI (OR 1.04; 95% CI 1.01, 1.08, *p* = 0.007) and can improve other predictive models that predict fatigue, which are based on severity. More precisely, every increase of 1 pg/mL IL-8 results in a 4% increased likelihood of experiencing fatigue 12 months after injury. However, they noted that IL-8 is also produced outside of the brain, and it is a non-specific marker of inflammation [38].

Liu et al. [40], in their study on using sodium valproate early in children with TBI, also observed high serum levels of IL-1β a day after admission (*p* < 0.017). Their study involved 45 children (30 with TBI), and they concluded that early use of sodium valproate can reduce both IL-1β and other markers of inflammation and improve prognosis (*p* < 0.05).

Lele et al. [41], in a study of 28 children with mild (GCS = 13–15), moderate (GCS = 9–12), and severe (GCS = 3–8) TBI, recorded plasma values of IL-6 and other markers (angiopoietin-2, endothelin-1, and endocan-2). They categorized children into four age groups: (1) 0–4 years, (2) 5–9 years, (3) 10–14 years, and (4) 15–18 years. These groups were then further divided into extra-axial and intra-axial hemorrhage, and finally, all four parameters were recorded once per day for 10 days after admission. For IL-6 (median = 23.37; range 6.26–180.54 pg/mL), no statistically significant difference was recorded for any period of time (*p* < 0.05); however, compared to mild and moderate TBI, those with sTBI had higher levels of IL-6 through day 10 after admission. The researchers also observed that IL-6 may be indicative of TBI severity, given that the biomarker patterns that they recorded suggest an inverse relationship between GCS and IL-6, although this was not statistically proven (*p* < 0.05). Finally, they noted that the type of lesion does not affect the biomarker level (*p* < 0.05) [41].

Chiaretti et al. [28] studied 29 children with sTBI (GCS ≤ 8), in which they measured CSF levels of IL-6 at two and four hours after injury. IL-6 levels increased 10-fold 2 h post-injury (*p* < 0.01) and then declined, although the latter observation was not significant (*p* = 1.17). They also reported no significant correlation between levels of IL-6 and GCS scores (*p* = 0.13), and they noted that the concurrent up-regulation of both IL-6 and NGF in the CSF can be a better predictor of neurological outcomes in patients with TBI (*p* < 0.01). However, the researchers remain skeptical because not only has previous research yielded controversial results, but the same team, in a previous study, also reported that IL-6 levels are associated with both severity and neurological outcomes in children with TBI [25]. Finally, apart from IL-6, they also correlate high levels of IL-1β with both severity and poor clinical outcomes in children with TBI [28]. Table 2 summarizes data from recent clinical studies.

## 6. ILs in Specific TBI-Related Clinical Conditions

### 6.1. ILs and Concussion

Serum biomarkers could be useful in cases of pediatric concussions, which account for 18% of all head injuries, and can have both short- and long-term effects [42]. The main clinical manifestations of concussions include headache, dizziness, difficulty in concentrating, cognitive or emotional impairment, and behavioral disturbances, while they can also lead to educational and emotional difficulties and have an impact on the overall quality of life. The mean recovery time for children and adolescents post-concussion is up to four weeks, even though approximately 30% of adolescents will present symptoms for more than one month post-injury [43].

Therefore, blood biomarkers could be used as predictors or indicators of post-concussion syndrome and could serve as both a diagnostic measure of severity and as a prognostic factor for clinical outcomes. In children with persisting symptoms indicative of concussion, levels of IL-6 and IL-8 vary significantly with time. IL-6 expression is high in this pediatric population. High concentration of IL-8 has also been associated with persisting symptoms, and its production is regulated by TNFα [44].

### 6.2. ILs and Intracranial Hypertension

According to what was described earlier, IL levels tend to rise in the CSF of children with TBI. Among the ILs that were described, IL-1β mostly tended to predict increased ICP in children with TBI, as described by Hayakata et al. [45], who found a positive correlation between CSF IL-1β and intracranial hypertension (r^2^ = 0.260, *p* < 0.05). Furthermore, results regarding IL-6 were also encouraging. As seen earlier, Hernegroeder et al. [29] showed that in children with isolated TBI, IL-6 could predict increased ICP.

### 6.3. IL-1β and Post-Traumatic Epilepsy

Post-traumatic epilepsy (PTE) is a leading cause of acquired epilepsy in youth, and it usually appears weeks to months following brain injury. Approximately 20% of patients with sTBI will develop PTE [46]. According to a large cohort study, including patients with moderate-to-severe TBI, both IL-1β and a specific IL-1β polymorphism have been correlated with PTE [47]. IL-1β is a pro-inflammatory cytokine that is increased after TBI, indicating a pro-inflammatory state with neuronal dysfunction. Apart from IL-1β, single-nucleotide polymorphisms (SNPs) in the gene responsible for IL-1β expression show a genetic susceptibility to PTE. More specifically, a high CSF/serum IL-1β concentration ratio (higher CSF/serum IL-1β ratio predicted PTE, PTE = 3.794 ± 2.274; no-PTE = 0.8207 ± 0.1495; *p* = 0.020) and a specific heterozygous cytosine–thymine genotype in one SNP of IL-1β (47.7% of such patients developed PTE, *p* = 0.016) are correlated with higher PTE risk. Interestingly, the combination of low serum concentration and high CSF concentration of IL-1β appears to have the strongest correlation with PTE. This biomarker could be very useful in designing an antiepileptic treatment strategy in TBI patients [47].

## 7. Clinical Implications and Future Directions

Recent advances in biomarker research have improved our understanding of the neuroinflammatory response in children with TBI [5]. Several factors need to be taken into account when analyzing cytokine data, as well as when designing such study protocols. For instance, the method of CSF drainage affects concentrations of ILs and other biomarkers in CSF. Intermittent drainage of CSF has been associated with nearly two-fold greater CSF concentrations of IL-6 than continuous drainage [8,48], which highlights the need for randomized studies comparing the two drainage techniques. This will, in turn, lead to standardized protocols for cytokine measurement [49].

In our analysis, a considerable number of studies measured IL levels in plasma, whereas fewer studies assessed IL levels in CSF. Notably, in a study of Ryan et al. [39], where IL-6 was measured in the plasma of pediatric TBI patients, a significant association between its levels and injury severity was found. On the other hand, Chiaretti et al. [28] found no such association when measuring IL-6 levels in the CSF. It is important to note, however, that the former study included mainly patients with mTBI, while in the latter, the authors recruited solely sTBI patients. It is, therefore, hypothesized that injury severity may also alter IL expression. For instance, Ryan et al. [39] showed that IL-6 levels and the IL-6/IL-8 ratio could predict sTBI. This was also supported by Lele et al. [41], who claimed that plasma IL-6 levels remained significantly elevated 10 days post-injury in patients with sTBI only.

Apart from methodological issues that need to be addressed, combining clinical and biochemical data may increase diagnostic and prognostic accuracy in pediatric TBI. The combination of post-resuscitation summated GCS and serum biomarker levels, especially serum IL-8 on day one, seems to be the best predictor for unfavorable outcomes when compared with GCS alone as an individual biomarker [12]. Findings also demonstrate the possible role of IL-1 as a biomarker of astrogliosis and, therefore, seizure susceptibility after TBI [49]. Additionally, as observed by Ryan et al. [39], IL-6 levels and the IL-6/IL-8 ratio helped in the diagnosis of pediatric patients with sTBI, so it could potentially help in managing these patients more effectively. Apart from that, we saw that several cytokine profiles were correlated with post-TBI symptoms, as IL-8 was linked to post-TBI fatigue [38] and IL-1β to post-TBI epilepsy and the need for pharmacological treatment [47].

Although the current findings are encouraging, reliable biomarkers for pediatric TBI are still lacking. Importantly, in adult TBI, molecules such as glial fibrillary acid protein (GFAP) and ubiquitin carboxy-terminal hydrolase L1 (UCH-L1) have recently been approved by the Food and Drug Administration (FDA) as blood biomarkers in mTBI [42]. These molecules, in addition to ILs and other chemokines such as NSE, NGF, BDNF, and S100B, could potentially be used in children, taking into account the specific reference values for each age group. Precisely, both groups of molecules are elevated in the serum of adult TBI patients shortly after the injury and are associated with injury severity, morbidity, mortality, and the need for acute neurosurgical intervention [50,51,52]. In studies assessing the long-term outcomes of mTBI, it is currently uncertain whether they can predict impaired recovery, as there are a number of studies that support an association between higher biomarker levels and worse long-term outcomes [53,54], while, on the contrary, some others describe only a modest association [55,56]. Apart from that, these biomarkers have been shown to predict computed tomography (CT) and magnetic resonance imaging (MRI) lesions and, thus, help in identifying the subset of patients who may benefit from them [57,58,59]. Notably, researchers from France and Spain created an automated blood test using cutoff values for GFAP and UCH-L1 and reported that it predicted the absence of intracranial lesions in patients with mTBI [60]. Such efforts may further increase diagnostic accuracy while simultaneously reducing unnecessary radiation exposure in selected patients.

There is also experimental evidence to support that inhibiting pro-inflammatory cytokines, either through receptor antagonism, downregulation, or antibody-mediated approaches, decreases neuronal loss and improves functional outcome after TBI [5,61,62,63]. As extracted from the above analysis, cytokines seem to play a crucial role in the CNS inflammatory response following TBI. Moreover, variability in cytokine responses and, thus, the degree of neuroinflammation post-TBI may be partly determined by gene polymorphisms within cytokine-encoding genes. Understanding these cytokine gene polymorphisms could help not only determine prognosis but also design targeted future management plans [7].

Finally, although cytokine level elevations are currently described, the magnitude of this increase is weaker in TBI when compared to other cases of inflammation, such as septic shock. This suggests that the profile of CNS cytokine levels in head trauma is unique, and there seems to be an imbalance between pro-inflammatory and anti-inflammatory cytokines [64]. Future studies should aim to investigate these differences and better describe cytokine interaction networks. This way, management options aiming to attenuate the neuroinflammatory cascade may be developed, ensuring disease-specific treatments for children undergoing head trauma.

## 8. Conclusions

To conclude, our analysis showed that ILs, mainly IL-1β, IL-6, and IL-8, play a role in regulating the neuroinflammatory cascade that follows acute injury in pediatric TBI, exhibiting pro- or anti-inflammatory effects, and affecting the expression and potency of other cytokines as well. Clinically, they can also be helpful, along with clinical and imaging criteria, in predicting sTBI forms and TBI-related clinical conditions, as well as the outcomes of these patients. Future research should aim to unravel the unique inflammatory pathways that are activated following TBI and to develop treatment strategies based on these mechanisms.

## Figures and Tables

**Table 1:** Summary of the main ILs in pediatric TBI.

Interleukin	Onset of Action	Expressed by	Action	Association with TBI Severity	Clinical Outcome Association
IL-1β	Moderate increase in the first two hours, remains high after 48 h [17]	Microglia, astrocytes, endothelial cells [11,22]	Pro-inflammatory [11,22,24]	Early rise was associated with severe head injury [11,22]	Poor [11,22]
IL-6	Rapid increase followed by decline after 48 h [17]	Astrocytes, neurons [26]	Anti-inflammatory [26]	Mixed results [9,25,26,27]	Mixed results, mainly favorable outcomes [11,17,28]
IL-8	Marked increase in the first 12 h [33]	Microglia, macrophages, endothelial cells [31]	Mixed (chemotactic, neutrophil-activating, neuroprotective) [32,33]	Associated with higher mortality [8,33]	Predicts post-injury fatigue [38]

IL, interleukin; TBI, traumatic brain injury.

**Table 2:** Summary of key recent clinical studies.

Authors	Year	Study Design	Type of Specimen	No of Patients	ILs Analyzed	Primary Findings	Secondary Findings
Ryan et al. [39]	2022	Prospective, controlled	Plasma	208	IL-2, IL-4, IL-6, IL-8, IL-10, and IL-17A	IL-6 was increased in every patient with TBI, and could distinguish mTBI from sTBI	IL-6/IL-8 ratio was the best marker regarding the differential diagnosis of sTBI from mTBI
Crichton et al. [38]	2021	Prospective, uncontrolled	Serum	87	IL-6 and IL-8	IL-8 levels can predict post-injury fatigue	-
Liu et al. [40]	2023	Prospective, controlled	Serum	45	IL-1β	High IL-1β levels upon submission	Sodium valproate can reduce inflammation and improve prognosis
Lele et al. [41]	2009	Prospective, uncontrolled	Plasma	28	IL-6	Patients with sTBI had higher IL-6 levels at day 10 compared to mTBI	Higher IL-6 levels tended to occur in patients with lower GCS
Chiaretti et al. [28]	2008	Prospective, controlled	CSF	29	IL-1β, IL-6	IL-1β correlated with severity and poor outcome, no association for IL-6	No association between IL-6 levels and GCS

GCS, Glasgow Coma Scale; CSF, cerebrospinal fluid; IL, interleukin; mTBI, mild traumatic brain injury; sTBI, severe traumatic brain injury; No, number.

## Data Availability

No datasets were generated for this study.

## Abbreviations

The following abbreviations are used in this manuscript:

AUC	Area under the curve
BBB	Blood–brain barrier
BDNF	Brain-derived neurotrophic factor
CBF	Cerebral blood flow
CNS	Central nervous system
CSF	Cerebrospinal fluid
CT CXCL8	Computed tomography C-X-C chemokine ligand family
ELISA	Enzyme-linked immunosorbent assay
INFγ	Interferon gamma
GCS	Glasgow coma scale
GDNF	Glial-derived neurotrophic factor
GFAP	Glial fibrillary acid protein
ICP	Intracranial pressure
IL	Interleukin
Jak1/Stat3	Janus kinase-signal transducer and activator of transcription
MAPK	Mitogen-activated protein kinase
MRI mTBI	Magnetic resonance imaging Mild traumatic brain injury
NFκB	Nuclear factor kappa-B
NGF	Nerve growth factor
NSE	Neuron specific enolase
PI3K	Phosphoinositide 3-kinase
PTE	Post-traumatic epilepsy
ROS	Reactive oxygen species
S100B	S100 calcium binding protein B
sNCAM	Soluble neural cell adhesion molecule
SNP	Single-nucleotide polymorphism
SOCS	Suppressor of cytokine signaling
sTBI	Severe traumatic brain injury
TBI	Traumatic brain injury
TNFα	Tumor necrosis factor alpha
UCH-L1	Ubiquitin carboxy-terminal hydrolase L1

## References

[1] de Souza L.C., Mazzu-Nascimento T., de Almeida Ballestero J.G., de Oliveira R.S., Ballestero M. (2023). Epidemiological study of paediatric traumatic brain injury in Brazil. World Neurosurg. X.

[2] Maas A.I.R., Menon D.K., Adelson P.D., Andelic N., Bell M.J., Belli A., Bragge P., Brazinova A., Büki A., Chesnut R.M. (2017). Traumatic brain injury: Integrated approaches to improve prevention, clinical care, and research. Lancet Neurol..

[3] Manley G.T., Dams-O’Connor K., Alosco M.L., Awwad H.O., Bazarian J.J., Bragge P., Corrigan J.D., Doperalski A., Ferguson A.R., Mac Donald C.L. (2025). A new characterisation of acute traumatic brain injury: The NIH-NINDS TBI classification and nomenclature initiative. Lancet Neurol..

[4] Thapa K., Khan H., Singh T.G., Kaur A. (2021). Traumatic Brain Injury: Mechanistic Insight on Pathophysiology and Potential Therapeutic Targets. J. Mol. Neurosci..

[5] Buttram S.D., Wisniewski S.R., Jackson E.K., Adelson P.D., Feldman K., Bayir H., Berger R.P., Clark R.S.B., Kochanek P.M. (2007). Multiplex assessment of cytokine and chemokine levels in cerebrospinal fluid following severe pediatric traumatic brain injury: Effects of moderate hypothermia. J. Neurotrauma.

[6] Corps K.N., Roth T.L., McGavern D.B. (2015). Inflammation and neuroprotection in traumatic brain injury. JAMA Neurol..

[7] Kalra S., Malik R., Singh G., Bhatia S., Al-Harrasi A., Mohan S., Albratty M., Albarrati A., Tambuwala M.M. (2022). Pathogenesis and management of traumatic brain injury (TBI): Role of neuroinflammation and anti-inflammatory drugs. Inflammopharmacology.

[8] Papa L., Ramia M.M., Kelly J.M., Burks S.S., Pawlowicz A., Berger R.P. (2013). Systematic review of clinical research on biomarkers for pediatric traumatic brain injury. J. Neurotrauma.

[9] Dewan M.C., Mummareddy N., Wellons J.C., Bonfield C.M. (2016). Epidemiology of global pediatric traumatic brain injury: Qualitative review. World Neurosurg..

[10] Park S.H., Hwang S.K. (2018). Prognostic Value of Serum Levels of S100 Calcium-Binding Protein B, Neuron-Specific Enolase, and Interleukin-6 in Pediatric Patients with Traumatic Brain Injury. World Neurosurg..

[11] Waters R.J., Murray G.D., Teasdale G.M., Stewart J., Day I., Lee R.J., Nicoll J.A.R. (2013). Cytokine gene polymorphisms and outcome after traumatic brain injury. J. Neurotrauma.

[12] Lo T.Y., Jones P.A., Minns R.A. (2010). Combining coma score and serum biomarker levels to predict unfavorable outcome following childhood brain trauma. J. Neurotrauma.

[13] Page M.J., McKenzie J.E., Bossuyt P.M., Boutron I., Hoffmann T.C., Mulrow C.D., Shamseer L., Tetzlaff J.M., Akl E.A., Brennan S.E. (2021). The PRISMA 2020 statement: An updated guideline for reporting systematic reviews. BMJ.

[14] Werner C., Engelhard K. (2007). Pathophysiology of traumatic brain injury. Br. J. Anaesth..

[15] Serpa R.O., Ferguson L., Larson C., Bailard J., Cooke S., Greco T., Prins M.L. (2021). Pathophysiology of Pediatric Traumatic Brain Injury. Front. Neurol..

[16] Roberto M., Patel R.R., Bajo M. (2018). Ethanol and Cytokines in the Central Nervous System. Handb. Exp. Pharmacol..

[17] Chiaretti A., Antonelli A., Riccardi R., Genovese O., Pezzotti P., Di Rocco C., Tortorolo L., Piedimonte G. (2008). Nerve growth factor expression correlates with severity and outcome of traumatic brain injury in children. Eur. J. Paediatr. Neurol..

[18] Jackman N.A., Hewett S.J., Claycomb R.J. (2012). Interleukin-1β in Central Nervous System Injury and Repair. Eur. J. Neurodegener. Dis..

[19] Erta M., Quintana A., Hidalgo J. (2012). Interleukin-6, a major cytokine in the central nervous system. Int. J. Biol. Sci..

[20] Zhang Y., Kong Q., Fan Y., Zhao H. (2025). Interleukin-2 and its receptors: Implications and therapeutic prospects in immune-mediated disorders of central nervous system. Pharmacol. Res..

[21] Giron S.E., Bjurstrom M.F., Griffis C.A., Ferrante F.M., Wu I.I., Nicol A.L., Grogan T.R., Burkard J.F., Irwin M.R., Breen E.C. (2018). Increased Central Nervous System Interleukin-8 in a Majority Postlaminectomy Syndrome Chronic Pain Population. Pain Med..

[22] Ozen I., Ruscher K., Nilsson R., Flygt J., Clausen F., Marklund N. (2020). Interleukin-1 Beta Neutralization Attenuates Traumatic Brain Injury-Induced Microglia Activation and Neuronal Changes in the Globus Pallidus. Int. J. Mol. Sci..

[23] Takahashi J.L., Giuliani F., Power C., Imai Y., Yong V.W. (2003). Interleukin-1beta promotes oligodendrocyte death through glutamate excitotoxicity. Ann. Neurol..

[24] Fogal B., Hewett S.J. (2008). Interleukin-1beta: A bridge between inflammation and excitotoxicity?. J. Neurochem..

[25] Chiaretti A., Genovese O., Aloe L., Antonelli A., Piastra M., Polidori G., Di Rocco C. (2005). Interleukin 1beta and interleukin 6 relationship with paediatric head trauma severity and outcome. Childs Nerv. Syst..

[26] Gruol D.L. (2015). IL-6 regulation of synaptic function in the CNS. Neuropharmacology.

[27] Bell M.J., Kochanek P.M., Doughty L.A., Carcillo J.A., Adelson P.D., Clark R.S., Wisniewski S.R., Whalen M.J., DeKosky S.T. (1997). Interleukin-6 and interleukin-10 in cerebrospinal fluid after severe traumatic brain injury in children. J. Neurotrauma.

[28] Chiaretti A., Antonelli A., Mastrangelo A., Pezzotti P., Tortorolo L., Tosi F., Genovese O. (2008). Interleukin-6 and nerve growth factor upregulation correlates with improved outcome in children with severe traumatic brain injury. J. Neurotrauma.

[29] Hergenroeder G.W., Moore A.N., McCoy J.P., Samsel L., Ward N.H., Clifton G.L., Dash P.K. (2010). Serum IL-6: A candidate biomarker for intracranial pressure elevation following isolated traumatic brain injury. J. Neuroinflamm..

[30] Tsitsipanis C. (2023). Molecular Biomarkers in Traumatic Brain Injury. Ph.D. Thesis.

[31] Kushi H., Saito T., Makino K., Hayashi N. (2003). IL-8 is a key mediator of neuroinflammation in severe traumatic brain injuries. Acta Neurochir. Suppl..

[32] Kossmann T., Stahel P.F., Lenzlinger P.M., Redl H., Dubs R.W., Trentz O., Schlag G., Morganti-Kossmann M.C. (1997). Interleukin-8 released into the cerebrospinal fluid after brain injury is associated with blood-brain barrier dysfunction and nerve growth factor production. J. Cereb. Blood Flow Metab..

[33] Whalen M.J., Carlos T.M., Kochanek P.M., Wisniewski S.R., Bell M.J., Clark R.S., DeKosky S.T., Marion D.W., Adelson P.D. (2000). Interleukin-8 is increased in cerebrospinal fluid of children with severe head injury. Crit. Care Med..

[34] Strle K., Zhou J.H., Shen W.H., Broussard S.R., Johnson R.W., Freund G.G., Dantzer R., Kelley K.W. (2001). Interleukin-10 in the brain. Crit. Rev. Immunol..

[35] Maiti P., Peruzzaro S., Kolli N., Andrews M., Al-Gharaibeh A., Rossignol J., Dunbar G.L. (2019). Transplantation of mesenchymal stem cells overexpressing interleukin-10 induces autophagy response and promotes neuroprotection in a rat model of TBI. J. Cell Mol. Med..

[36] Waisman A., Hauptmann J., Regen T. (2015). The role of IL-17 in CNS diseases. Acta Neuropathol..

[37] Kostic M., Zivkovic N., Cvetanovic A., Stojanovic I., Colic M. (2017). IL-17 signalling in astrocytes promotes glutamate excitotoxicity: Indications for the link between inflammatory and neurodegenerative events in multiple sclerosis. Mult. Scler. Relat. Disord..

[38] Crichton A., Ignjatovic V., Babl F.E., Oakley E., Greenham M., Hearps S., Delzoppo C., Beauchamp M.H., Guerguerian A.M., Boutis K. (2021). Interleukin-8 Predicts Fatigue at 12 Months Post-Injury in Children with Traumatic Brain Injury. J. Neurotrauma.

[39] Ryan E., Kelly L., Stacey C., Huggard D., Duff E., McCollum D., Leonard A., Boran G., Doherty D.R., Bolger T. (2022). Mild-to-severe traumatic brain injury in children: Altered cytokines reflect severity. J. Neuroinflamm..

[40] Liu Z., Zhu L., Sheng L.P., Huang Q.C., Qian T., Qi B.X. (2023). A pilot study on the effects of early use of valproate sodium on neuroinflammation after traumatic brain injury. Zhongguo Dang Dai Er Ke Za Zhi.

[41] Lele A.V., Alunpipatthanachai B., Qiu Q., Clark-Bell C., Watanitanon A., Moore A., Chesnut R.M., Armstead W., Vavilala M.S. (2019). Plasma Levels, Temporal Trends and Clinical Associations between Biomarkers of Inflammation and Vascular Homeostasis after Pediatric Traumatic Brain Injury. Dev. Neurosci..

[42] Veliz P., McCabe S.E., Eckner J.T., Schulenberg J.E. (2017). Prevalence of concussion among US adolescents and correlated factors. JAMA.

[43] Eisenberg M.A., Meehan W.P., Mannix R. (2014). Duration and course of post- concussive symptoms. Pediatrics.

[44] Parkin G.M., Clarke C., Takagi M., Hearps S., Babl F.E., Davis G.A., Anderson V., Ignjatovic V. (2019). Plasma Tumor Necrosis Factor Alpha Is a Predictor of Persisting Symptoms Post-Concussion in Children. J. Neurotrauma.

[45] Hayakata T., Shiozaki T., Tasaki O., Ikegawa H., Inoue Y., Toshiyuki F., Hosotubo H., Kieko F., Yamashita T., Tanaka H. (2004). Changes in CSF S100B and cytokine concentrations in early-phase severe traumatic brain injury. Shock.

[46] Park J.T., DeLozier S.J., Chugani H.T. (2021). Epilepsy Due to Mild TBI in Children: An Experience at a Tertiary Referral Center. J. Clin. Med..

[47] Diamond M.L., Ritter A.C., Failla M.D., Boles J.A., Conley Y.P., Kochanek P.M., Wagner A.K. (2014). IL-1β associations with posttraumatic epilepsy development: A genetics and biomarker cohort study. Epilepsia.

[48] Shore P.M., Thomas N.J., Clark R.S., Adelson P.D., Wisniewski S.R., Janesko K.L., Bayir H., Jackson E.K., Kochanek P.M. (2004). Continuous versus intermittent cerebrospinal fluid drainage after severe traumatic brain injury in children: Effect on biochemical markers. J. Neurotrauma.

[49] Semple B.D., O’Brien T.J., Gimlin K., Wright D.K., Kim S.E., Casillas-Espinosa P.M., Webster K.M., Petrou S., Noble-Haeusslein L.J. (2017). Interleukin-1 Receptor in Seizure Susceptibility after Traumatic Injury to the Pediatric Brain. J. Neurosci..

[50] Papa L., McKinley W.I., Valadka A.B., Newman Z.C., Nordgren R.K., Pramuka P.E., Barbosa C.E., Brito A.M.P., Loss L.J., Tinoco-Garcia L. (2024). Diagnostic performance of GFAP, UCH-L1, and MAP-2 Within 30 and 60 Minutes of traumatic brain injury. JAMA Netw. Open.

[51] Vos P.E., Jacobs B., Andriessen T.M., Lamers K.J., Borm G.F., Beems T., Edwards M., Rosmalen C.F., Vissers J.L. (2010). GFAP and S100B are biomarkers of traumatic brain injury: An observational cohort study. Neurology.

[52] Posti J.P., Takala R.S., Runtti H., Newcombe V.F., Outtrim J., Katila A.J., Frantzén J., Ala-Seppälä H., Coles J.P., Hossain M.I. (2016). The levels of Glial Fibrillary Acidic Protein and Ubiquitin C-Terminal Hydrolase-L1 during the first week after a traumatic brain injury: Correlations with clinical and imaging findings. Neurosurgery.

[53] Whitehouse D.P., Wilson L., Czeiter E., Buki A., Wang K.K.W., von Steinbüchel N., Zeldovich M., Steyerberg E., Maas A.I.R., Menon D.K. (2025). Association of blood-based biomarkers and 6-Month patient-reported outcomes in patients with mild TBI: A CENTER-TBI analysis. Neurology.

[54] Godeiro Coelho L.M., Teixeira F.J.P., Koru-Sengul T., Manolovitz B., Taylor R.R., Massad N., Kottapally M., Merenda A., Munoz Pareja J.C., de Rivero Vaccari J.P. (2025). Predictive value of nervous cell injury biomarkers in moderate-to-severe traumatic brain injury: A network meta-analysis. Neurology.

[55] Korley F.K., Jain S., Sun X., Puccio A.M., Yue J.K., Gardner R.C., Wang K.K.W., Okonkwo D.O., Yuh E.L., Mukherjee P. (2022). Prognostic value of day-of-injury plasma GFAP and UCH-L1 concentrations for predicting functional recovery after traumatic brain injury in patients from the US TRACK-TBI cohort: An observational cohort study. Lancet Neurol..

[56] Behzadi F., Luy D.D., Schaible P.A., Zywiciel J.F., Puccio A.M., Germanwala A.V. (2024). A systematic review and meta-analysis of major blood protein biomarkers that predict unfavorable outcomes in severe traumatic brain injury. Clin. Neurol. Neurosurg..

[57] Hossain I., Marklund N., Czeiter E., Hutchinson P., Buki A. (2023). Blood biomarkers for traumatic brain injury: A narrative review of current evidence. Brain Spine.

[58] Karamian A., Farzaneh H., Khoshnoodi M., Maleki N., Rohatgi S., Ford J.N., Romero J.M. (2025). Accuracy of GFAP and UCH-L1 in predicting brain abnormalities on CT scans after mild traumatic brain injury: A systematic review and meta-analysis. Eur. J. Trauma Emerg. Surg..

[59] Amoo M., Henry J., O’Halloran P.J., Brennan P., Husien M.B., Campbell M., Caird J., Javadpour M., Curley G.F. (2022). S100B, GFAP, UCH-L1 and NSE as predictors of abnormalities on CT imaging following mild traumatic brain injury: A systematic review and meta-analysis of diagnostic test accuracy. Neurosurg. Rev..

[60] Lagares A., de la Cruz J., Terrisse H., Mejan O., Pavlov V., Vermorel C., Payen J.F., BRAINI participants and investigators (2024). An automated blood test for glial fibrillary acidic protein (GFAP) and ubiquitin carboxy-terminal hydrolase L1 (UCH-L1) to predict the absence of intracranial lesions on head CT in adult patients with mild traumatic brain injury: BRAINI, a multicentre observational study in Europe. EBioMedicine.

[61] Lloyd E., Somera-Molina K., Van Eldik L.J., Watterson D.M., Wainwright M.S. (2008). Suppression of acute proinflammatory cytokine and chemokine upregulation by post-injury administration of a novel small molecule improves long-term neurologic outcome in a mouse model of traumatic brain injury. J. Neuroinflamm..

[62] Bachstetter A.D., Webster S.J., Goulding D.S., Morton J.E., Watterson D.M., Van Eldik L.J. (2015). Attenuation of traumatic brain injury-induced cognitive impairment in mice by targeting increased cytokine levels with a small molecule experimental therapeutic. J. Neuroinflamm..

[63] Bachstetter A.D., Zhou Z., Rowe R.K., Xing B., Goulding D.S., Conley A.N., Sompol P., Meier S., Abisambra J.F., Lifshitz J. (2016). MW151 inhibited IL-1β levels after traumatic brain injury with no effect on microglia physiological responses. PLoS ONE.

[64] Bell M.J., Kochanek P.M., Doughty L.A., Carcillo J.A., Adelson P.D., Clark R.S.B., Whalen M.J., DeKosky S.T. (1997). Comparison of the interleukin-6 and interleukin-10 response in children after severe traumatic brain injury or septic shock. Acta Neurochir. Suppl..

